# A mitochondrial strategy for safeguarding the reprogrammed genome

**DOI:** 10.1186/2045-9769-3-5

**Published:** 2014-03-29

**Authors:** Alessandro Prigione, James Adjaye

**Affiliations:** 1 Max Delbrueck Center for Molecular Medicine (MDC), Robert-Roessle-Str. 10, D-13125 Berlin-Buch, Germany; 2 Department of Vertebrate Genomics, Molecular Embryology and Aging Group, Max Planck Institute for Molecular Genetics, Berlin, Germany; 3 Institute for Stem Cell Research and Regenerative Medicine, Heinrich Heine University, Duesseldorf, Germany

**Keywords:** Antioxidants, Mitochondria, Reprogramming, iPSCs, Genomic aberrations

## Abstract

Genomic aberrations induced by somatic cell reprogramming are a major drawback for future applications of this technology in regenerative medicine. A new study by Ji et al. published in Stem Cell Reports suggests a counteracting strategy based on balancing the mitochondrial/oxidative stress pathway through antioxidant supplementation.

## Commentary

Reactive oxygen species (ROS) are common by-products of cellular respiration. They can act as second messengers exerting physiological roles [1]. However, if ROS levels increase beyond a certain threshold, functional oxidative damage to macromolecules can occur, leading to protein, lipid or genomic aberrations and eventually cell death [2]. To preserve genome integrity, cells have developed a fine-tuned machinery to counteract ROS by keeping them in equilibrium with reducing equivalents [1, 2]. The maintenance of redox balance is thus critical for cells both in steady states and during adaptations to different conditions. Now, a new study by Ji et al. [3] demonstrates that supporting redox homeostasis is important also during the induction of pluripotency.

The authors detected increased levels of ROS and oxidative DNA damage during the early stages of human retroviral-based reprogramming using four factors (4F: OCT4, SOX2, KLF4, c-MYC), in agreement with previous reports [4, 5]. Notably, the concurrent supply of antioxidants (vitamin C or N-acetyl-cysteine, NAC) appeared capable of reducing both ROS and genomic double-strand breaks, resulting in lower apoptotic rates. These effects were not a consequence of altered transgene activity, since antioxidants did not modify the 4F expression or their silencing. Remarkably, induced pluripotent stem cells (iPSCs) lines generated with antioxidant supplementation displayed significantly fewer *de novo* copy number variations (CNVs), i.e. genomic variants that were not already present in the parental fibroblast population. To rule out that the reduction in the number of CNVs was not due to additional non-antioxidant related mechanisms influencing reprogramming, which have been found associated with vitamin C supplementation [6, 7], the authors demonstrated that CNVs were similarly lowered by vitamin C and NAC treatment. It must also be noted that culture media typically employed for human reprogramming (e.g. KSR and mTeSR) contains vitamin C, suggesting that in its absence the levels of ROS would be higher. Hence, supporting the redox balance through the addition of reducing molecules may protect the somatic genome, leading to iPSCs with fewer genomic alterations.

**Figure 1:**
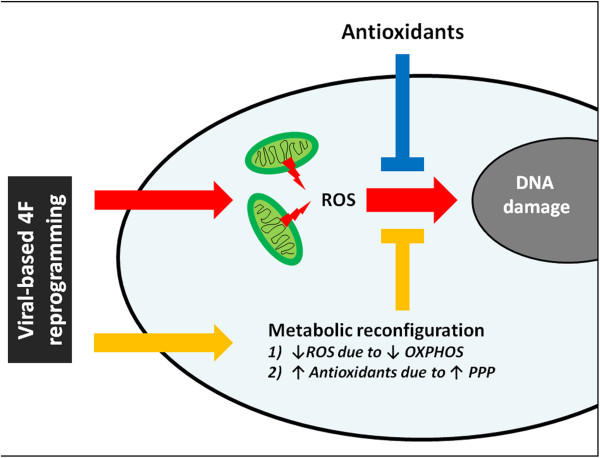
**The induction of pluripotency involves the establishment of a cellular state characterized by low levels of oxidative stress.** This is accomplished through a global metabolic restructuring which leads to reduced mitochondrial oxidative phosphorylation (OXPHOS) and increased energy flux towards glycolysis and the pentose phosphate pathway (PPP) (orange arrows). However, this glycolytic shift is not adequately sufficient to prevent the increased leakage of reactive oxygen species (ROS) associated with viral-based four factor (4F) reprogramming. This results in DNA damage that may have detrimental consequences on iPSC functionality (red arrows). The paper by Ji et al. demonstrates that the supplementation with ROS-scavenging molecules provides additional defense against redox imbalance, giving rise to iPSCs bearing fewer genomic aberrations (blue arrows).

Reprogramming somatic cells to pluripotency involves a profound cellular reconfiguration associated with high proliferative rates and a shift towards glycolysis-based metabolism even in the presence of oxygen [8, 9], a situation reminiscent of the Warburg effect that occurs upon cancer transformation. Maintaining the redox equilibrium would therefore be essential for cells undergoing such dramatic restructuring. Indeed, tumor cells re-route the energy flux outside the mitochondria and into the pentose phosphate pathway (PPP) in order to provide important reducing equivalents and diminish the generation of mitochondrial ROS. In this regard, recent evidence suggests that a central player in cancer is pyruvate kinase isoform M2 (PKM2), increased levels of which lead to higher concentrations of glucose-6-phosphate (G6P) and enhanced PPP activity [10]. Interestingly, PKM2 and G6P are also up-regulated upon the induction of pluripotency [11, 12], indicating that similar mechanisms aiming at maintaining redox homeostasis are also in place during reprogramming. Supporting this idea, increased ROS levels promote the differentiation of stem cells [13] and low ROS levels are a characteristic of undifferentiated pluripotent stem cells [8, 14]. However, in agreement with previous observations [4, 5], the work of Ji et al. [3] demonstrates that the redox equilibrium is not sufficiently balanced during reprogramming (Figure 1).

Their findings also raise a series of important questions. For example, how is it that somatic-coding mutations are not affected by the introduction of antioxidants? The authors suggest that oxidative DNA lesions might be less error-prone and therefore more easily corrected. Moreover, is it possible to employ additional conditions that potentiate the effects of the antioxidant cocktail? In this regard, hypoxia or the addition of a hypoxia mimetic might be beneficial, given that hypoxia enhances iPSC derivation [15], by inducing a faster glycolytic transition [12]. Likewise, do antioxidants protect against mitochondrial mutations acquired during reprogramming [11]?

Another central issue that remains to be addressed is the relationship between antioxidant supplementation, reprogramming methods and genomic aberrations. Although mutations have been found to occur also using non-integrating strategies [16], the levels of nuclear and mtDNA alterations may be diminished under these conditions [17]. Indeed, non-integrating episomal plasmids elicit a lower ROS response than viral-based reprogramming [5]. A systematic comparison using various iPSC techniques with and without antioxidant treatment would help to clarify this matter.

Finally, the data by Ji et al. [3] underscores the unique features of c-MYC within the 4F cocktail. c-MYC is a key inducer of glycolytic reconfiguration [18] but also appears as the major contributor of reprogramming-mediated oxidative stress. In fact, the use of the other three factors did not generate a drastic elevation of ROS nor was their basal level affected by antioxidant supplementation [3]. Nonetheless, genomic aberrations and metabolic conversion can occur also in the absence of c-MYC [19, 20]. Hence, reprogramming strategies should ideally avoid the inclusion of c-MYC, and it remains unclear whether such strategies would also benefit from the addition of antioxidants.

Overall, the work by Ji et al. [3] has relevant implications, as the occurrence of reprogramming-mediated genomic alterations is currently a major obstacle hindering the use of iPSCs in medical applications [21]. Further manipulation of the mitochondrial/oxidative stress pathway may thus pave the way for the development of safer reprogramming approaches.
